# CCL20-CCR6 axis modulated traumatic brain injury-induced visual pathologies

**DOI:** 10.1186/s12974-019-1499-z

**Published:** 2019-05-31

**Authors:** Mahasweta Das, Xiaolan Tang, Jung Yeon Han, Karthick Mayilsamy, Elspeth Foran, Manas R. Biswal, Radouil Tzekov, Shyam S. Mohapatra, Subhra Mohapatra

**Affiliations:** 1 James A. Haley Veterans Hospital, Tampa, FL USA; 2 Department of Molecular Medicine, Morsani College of Medicine, University of South Florida, Tampa, FL USA; 3 Department of Internal Medicine, Morsani College of Medicine, University of South Florida, Tampa, FL USA; 4 Department of Ophthalmology, Morsani College of Medicine, University of South Florida, Tampa, FL USA; 5 Graduate Programs at College of Pharmacy, University of South Florida, Tampa, FL USA; 6 Department of Medical Engineering, University of South Florida, Tampa, FL USA; 7 The Roskamp Institute, Sarasota, FL USA

**Keywords:** rTBI, CCL20, Pioglitazone, CCR6−/−, Retina, RGC

## Abstract

**Background:**

Traumatic brain injury (TBI) is a major cause of death and disability in the USA and the world; it constitutes 30% of injury-related deaths (Taylor et al., MMWR Surveill Summ 66:1-16, 2017). Contact sports athletes often experience repetitive TBI (rTBI), which exerts a cumulative effect later in life. Visual impairment is a common after-effect of TBI. Previously, we have shown that C-C chemokine 20 (CCL20) plays a critical role in neurodegeneration and inflammation following TBI (Das et al., J Neuroinflammation 8:148, 2011). C-C chemokine receptor 6 (CCR6) is the only receptor that CCL20 interacts with. The objective of the present study was to investigate the role of CCL20-CCR6 axis in mediating rTBI-induced visual dysfunction (TVD).

**Methods:**

Wild type (WT) or CCR6 knock out (CCR6−/−) mice were subjected to closed head rTBI. Pioglitazone (PG) is a peroxisome proliferator-activated receptor γ (PPARγ) agonist which downregulates CCL20 production. Subsets of WT mice were treated with PG following final rTBI. A subset of mice was also treated with anti-CCL20 antibody to neutralize the CCL20 produced after rTBI. Histopathological assessments were performed to show cerebral pathologies, retinal pathologies, and inflammatory changes induced by rTBI.

**Results:**

rTBI induced cerebral neurodegeneration, retinal degeneration, microgliosis, astrogliosis, and CCL20 expression. CCR6−/− mice showed reduced retinal degeneration, microgliosis, and inflammation. Treatment with CCL20 neutralization antibody or PG showed reduced CCL20 expression along with reduced retinal degeneration and inflammation. rTBI-induced GFAP-positive glial activation in the optic nerve was not affected by knocking out CCR6.

**Conclusion:**

The present data indicate that rTBI-induced retinal pathology is mediated at least in part by CCL20 in a CCR6-dependent manner.

**Electronic supplementary material:**

The online version of this article (10.1186/s12974-019-1499-z) contains supplementary material, which is available to authorized users.

## Background

Traumatic brain injury (TBI) is one of the major causes of morbidity and mortality worldwide. Thirty percent of injury-related deaths are TBI-related [1]. Common causes of TBI include fall, motor vehicle accidents, sports, assaults, and battlefield injuries. TBI causes significant long-term deficits including behavioral and functional deficits [2], cognitive deficits [3], sensorimotor deficits [4], psychiatric disorders [5] and memory deficits [6], vision [7–9] and hearing deficits [10]. Based on the Glasgow Coma Scale scores, TBI can be categorized into mild (≤ 8), moderate (9–12), and severe (13–15) [11]. The mechanical injury caused by initial insult to the head causes cellular and tissue damage. This evokes systemic and local inflammatory responses, which lead to secondary neurodegeneration including long-term neurological, functional, cognitive, and behavioral deficits.

TBI affects the eye and the visual system. The prevalence of visual dysfunction after TBI varies from 30 to 85% [12]. Optic nerve injury is one of the most common events after TBI [13]. Between 2000 and 2013, an estimated 287,861 military personnel sustained TBI, most of which were classified as mild TBI. In a retrospective cohort study among US veterans, Lee et al. reported that 37.2% of TBI patients were suffering from dry eye disease (DED), suggesting a common underlying pathogenic mechanism [14]. In addition, corneal abrasion, blepharitis, chalazion/hordeolum, traumatic cataract, vitreal prolapse, and optic atrophy have been reported in TBI patients [15, 16]. Vien et al. reported trans-synaptic retrograde degeneration of retinal nerve fibers, progressive loss of retinal nerve fiber layer, and visual field loss in a patient 2 months after severe TBI. They also reported optic nerve thinning and retrograde ganglion cell loss secondary to TBI in this patient [17]. Tzekov et al. have recently shown that mild rTBI causes retinal ganglion cell (RGC) loss and optic nerve inflammation in a mouse model [8].

The C-C chemokine ligand 20 (CCL20) (also known as macrophage inflammatory protein-3α, MIP3α) is a small chemokine of about 8 kDa encoded by the SCYA20 gene located on chromosome 2 in humans [18]. Constitutive expression of this chemokine can be found in tissues of peripheral organs, such as the intestines, lung, liver, and lymphoid tissues (lymph nodes and Peyer’s patches) [19–22]. CCL20 has a sole C-C chemokine receptor type 6 (CCR6) [23]. The CCR6-CCL20 binding attracts immature dendritic cells (DC), effector or memory T cells and B cells [20] to the target organ. CCL20/CCR6 axis has been implicated in multiple disease processes in humans including diabetic nephropathy [24], lung fibrosis [25, 26], chronic liver disease [19, 27], DED [28], and inflammatory bowel disease [21]. Studies have shown that CCL20 plays an important role in neurodegeneration following TBI [29–31], spinal cord injury [32], and cerebral ischemia [33] in rodents. CCL20 has also been shown to be upregulated along with other cytokines in human plasma on the day after severe TBI [34]. This chemokine is constitutively expressed in brain choroid plexus of mouse and humans and binds with its sole receptor CCR6 expressed on T cells [35]. Available data shows that the binding of CCL20 to CCR6-positive Th17 cells is critical for T cell infiltration into the CNS through the choroid plexus [35]. Involvement of CCL20-CCR6 axis in ocular diseases has also been shown by several authors. DED is a major ocular surface disorder. Patients suffer from eye irritation, blurred vision, and light sensitivity which decrease their quality of life significantly [36]. Recently, Coursey et al. have shown the involvement of CCL20-CCR6 axis in development of DED [37]. Age-related macular degeneration (AMD) is a retinal degenerative disease leading to irreversible vision loss. Involvement of CCR6+ Th17 cells in blood samples of AMD patients have been indicated by Singh et al. [38]. Rossi et al. have shown CCL20 gene expression in the mouse retina following experimental diabetic retinopathy [39]. But the role of CCL20/CCR6 axis in the rTBI-induced visual dysfunction (TVD) has not been elucidated.

Previously, our laboratory has shown that TBI evokes CCL20 expression in the spleen 24 h after TBI and in the brain 48 h after TBI and splenectomy reduced the TBI-induced neurodegeneration and CCL20 expression in the brain [29, 31], indicating a role of systemic CCL20 signaling in TBI-induced neurodegeneration. In this study, we hypothesize that CCL20-CCR6 axis plays a critical role in TVD. This hypothesis has been tested in a well-established mouse model of mild rTBI using CCR6 knockout (CCR6−/−) mice as well as by pharmacologically blocking CCL20 using anti-CCL20 antibody and pioglitazone (PG). PG is a peroxisome proliferator-activated receptor γ (PPARγ) agonist belonging to thiazolidinedione (TZD) class of drugs. This drug is used mainly to treat hyperglycemia. When used in low dose, it showed a cardio-protective property in experimental animals [40]. Qi et al. have shown that PPARγ negatively regulates CCL20 expression in the renal epithelium [41]. PG has also been shown to reduce proinflammatory cytokines IL1-β and IL-6 in different tissues [42–44]. Its neuroprotective effects following TBI have been shown by Sauerbeck et al. [45]. In this study, we have tested the efficacy of PG in mitigating rTBI-induced visual pathologies.

## Methods

### Animals, induction of mild rTBI, and drug delivery

All animal procedures in this study followed NIH guidelines and were approved by the Institutional Animal Care and Use Committee of University of South Florida. Adult 14–16 weeks old, male C57BL/6 or CCR6−/− mice (Jackson laboratory, Bar Harbor, ME) were housed in the animal facility at 12 h light–12 h dark cycle with food and water available *ad libitum*. Mice were anesthetized with continuous flow of a mixture of 2% isoflurane and oxygen and placed on a heating pad to maintain body temperature. Mice heads were fixed in a stereotaxic instrument (Model no. 900, David Kopf instruments, CA). The skin on the head covering the impact area and about 1 cm margin around it was shaved. A controlled cortical injury device (CCI, Impact One Stereotaxic Impactor, Leica Biosystems, Buffalo Grove, IL) was positioned at an antero-posterior (AP) coordinate of − 0.8 mm from the bregma and on the midline of the skull. Impact was delivered by a metal impactor with a tip diameter of 5 mm, striking velocity 5 m/s, striking depth of 1.5 mm, and 200 ms dwell time. Following the impact, the mouse was allowed to recover from anesthesia on a heating pad. When sternal recumbence was regained, the mouse was returned to the home cage. Five hits were delivered at an interval of 48 h between each hit [7, 8]. No skull fracture, hemorrhages, or cerebral contusions occurred in this method. Sham mice received anesthesia of equal duration to that of rTBI mice every 48 h and were placed on the stereotaxic frame, but no CCI was delivered. Mice were divided into the following experimental groups: sham = 5, rTBI = 9, rTBI + vehicle = 5, rTBI + PG = 5, CCR6−/− sham = 6, CCR6−/− rTBI = 9, rTBI + isotype = 4, rTBI + anti-CCL20 antibody = 4.

Pioglitazone hydrochloride (PG) (Tocris Bioscience, Bristol, UK) was dissolved in dimethylsulfoxide (DMSO). Two hours after the last rTBI induction, mice were given a single intraperitoneal injection of PG. After initial optimization, PG was injected at a dose of 10 mg/kg in a volume of 40 μL per mouse. Equal volume of DMSO was injected in the vehicle group.

In a subset of animals, rTBI was induced as described above. Following each TBI, 20 μg of mouse monoclonal IgG (MAB760, R&D systems) or isotype control (MAB005 R&D systems) was injected via i.p. route. Each group in this experiment had four mice.

### Euthanasia and tissue harvest

Seven days after the final injury (7 days post-injury, 7 dpi), the animals were deeply anesthetized with euthasol (150 μg/kg). Eyes and optic nerves were collected and post-fixed in 4% paraformaldehyde (PFA) for pathological analysis. Then, mice were transcardially perfused first with 0.1 M phosphate buffer and then with 4% PFA in phosphate buffer. Brains were harvested, post-fixed overnight with 2% PFA. The brains and optic nerves were cryoprotected with 30% sucrose. Thirty-micrometer cryosections were produced from the brain, and 5-μm longitudinal cryosections were produced from the optic nerve for staining experiments. The eyes were infiltrated and embedded in paraffin, and 5-μm sections were produced for staining experiments.

### Fluoro-Jade staining

Thirty-micrometer brain sections were hydrated using graded alkaline ethanol starting with 100% and then decreasing to 70% and 50% and water for 1 min for each solution. Sections were then oxidized using 0.06% KMnO_4_ solution for 15 min followed by three rinses in dIH_2_O for 2 min each. Sections were then incubated with 0.001% solution of Fluoro-Jade in 0.1% acetic acid for 30 min on a slow rocker. Slides were rinsed, dried at 45 °C for 20 min, cleared with xylene, and cover-slipped using DPX mounting medium (Electron Microscopy Sciences, Ft. Washington, PA).

### Retinal flat mount preparation and retinal ganglion cell labeling

The enucleated eyes from each mouse were fixed in 4% paraformaldehyde overnight; the corneas were removed by cutting in a circular path along the ora serrata with small scissors, holding the eye at the limbus with forceps. The lenses were pulled out with forceps. Vitreous humor was removed as much as possible, and the retina was detached from the eyecup by positioning forceps between the retina and the eyecup, moving the forceps slowly around the circumference. After separation, the retina was permeated by freezing in 0.5% Triton X-100 in phosphate-buffered saline (PBS) for 15 min at −70 °C. The free-floating retinae were then washed in 0.5% Triton PBS three times for 10 min each and incubated with primary rabbit anti-RBPMS (RNA binding protein with multiple splicing) antibody (1:500, rabbit polyclonal, Abcam, cat. No. ab194213) overnight at 4 °C. Retinae were then washed three times for 10 min each with PBS and incubated with secondary antibody (Dylight594 Goat anti-rabbit 1: 1000, Thermo Fisher Scientific, cat No. A11008) at room temperature for 2 h. Retinae were then thoroughly washed in PBS and mounted with the vitreal side up on slides. To mount the entire retina flat on the slide, incisions were made from the periphery towards the center and then were cover-slipped with Vectashield anti-fade mounting medium solution with DAPI (Vector labs, Burlingame, Ca, USA).

### Hematoxylin and eosin (H&E) staining

Slide mounted, 5-μm thick paraffin sections of the eyes were deparaffinized with xylene and hydrated with graded ethanol with increasing water content and finally water alone. Hematoxylin and eosin stains were applied sequentially with three washes with dI H_2_O in between. Slides were dehydrated with graded ethanol with decreasing water content, cleared with xylene, and cover-slipped using DPX mounting medium. Images were viewed by Olympus BX51 microscope, and bright field images were taken using Olympus DP70 camera (Olympus America Inc., Center Valley, PA).

### Fluorescence immunohistochemistry

Thirty-micrometer-thick brain and 5-μm-thick optic nerve cryosections were washed with three changes of PBS. Slide-mounted paraffin sections were deparaffinized with two changes of xylene, each for 5 min followed by graded hydration in ethanol with increasing water content. Antigen retrieval was performed on the hydrated sections by heating them in antigen unmasking solution (Vector lab) diluted in PBS (1:100) for 30 min. The sections were cooled and washed with PBS three times. All the sections were blocked with 10% goat serum solution with 0.1% Triton X-100 in PBS for 1 h at room temperature. Sections were then incubated with chicken anti-glial fibrillary acidic protein (GFAP) (1:1000, chicken polyclonal, Millipore AB5541) or rabbit anti-ionized calcium binding adaptor molecule (Iba1) (1:500, rabbit polyclonal, Wako 019-19741) or rabbit anti-RBPMS (1:100, rabbit polyclonal, Abcam, ab 194213) diluted in antibody solution containing 5% serum and 0.5% Triton X-100 in PBS overnight at 4 °C in a moist incubation box. The sections were then washed in PBS three times each for 10 min. Sections were incubated with secondary antibodies for 2 h at room temperature. Secondary antibodies used in this study are Alexa Fluor 488-conjugated goat anti-chicken (1:1000, Abcam, ab150169), Alexa Fluor 488-conjugated goat anti-rabbit (1:1000, Vector labs DI-1594), and DyLight 594-conjugated goat anti-rabbit (1:1000, Thermo Fisher, A11008) IgGs. The sections were washed three times in PBS for 5 min each, dried, and cover-slipped using Vectashield anti-fade hardset mounting medium (Vector, H-1500). Sections were viewed with Olympus BX51 fluorescence microscope using appropriate filters, and images were taken with Olympus DP70 camera. Images were taken using same exposure and camera settings for a given antigen to minimize background fluorescence in control and experimental groups.

### Immunoperoxidase staining

Slide-mounted cryosections were washed with PBS, and antigen retrieval was performed as mentioned in the immunofluorescence staining section above. The sections were then treated with 3% hydrogen peroxide for 20 min and blocked with 10% goat serum with 0.1% Triton X-100 in PBS for 1 h at room temperature. Sections were incubated with rabbit-anti CCL20 antibody (1:1000, Thermo Fisher PA5-68465) or rabbit anti-CCR6 antibody (1:1000, rabbit polyclonal, Abcam ab78429) overnight at 4 °C in a moist incubation chamber. Sections were washed with three changes of PBS and incubated with biotinylated anti-rabbit secondary antibody (1:400, Vector labs) for 2 h at room temperature. After washing, the sections were incubated in ABC solution (Vector labs) for 30 min following the manufacturer’s instruction. Sections were developed with diaminobenzidine (DAB) solution. Sections were thoroughly washed in dI H_2_O, dried, cover-slipped with DPX mounting medium, and viewed with Olympus BX51 microscope. Bright field images were taken using Olympus DP72 camera.

### Ganglion cell counting

Images of retinal whole mount stained with anti RBPMS antibody were acquired by Keyence fluorescence microscope (BZ-X710, Keyence America, IL, USA) with × 20 objective using Texas Red filter. Images were then stitched together using Keyence stitching tool to reproduce the whole retina. RBPMS-positive cells were counted from the mid-peripheral zone of the reproduced image of the retina as shown by the box in Fig. 5b using Keyence image processing tool in masking mode. The RBPMS-positive cells were specified using mask separating individual cells. The total number of cells was then counted. This automatic counting procedure was validated in a subset of samples which showed excellent comparison with manual counting and therefore used in the entire study.

### Enzyme-linked immunosorbent assay

Snap-frozen mouse eyes were thawed in ice-cold lysis buffer, and retina was isolated under dissection microscope. The retinal tissue was homogenized and protein was extracted. Total protein concentration was measured using Bradford assay. Enzyme-linked immunosorbent assay (ELISA) was performed for detection of CCL20 in the tissue using Mouse CCL20/MIP-3 alpha Quantikine ELISA Kit (Cat # MCC200, R & D Systems, Minneapolis, MN) following the manufacturer’s instruction. Briefly, 50 μL assay diluent and 50 μL standard or samples were added to wells of a microplate pre-coated with monoclonal anti-mouse CCL20 antibody and incubated for 2 h at room temperature allowing thorough mixing. The wells were washed and incubated with monoclonal mouse CCL20 conjugated with horseradish peroxidase for 2 h at room temperature. The wells were treated with substrate reagent and read at 450 nm with correction at 540 nm using a Synergy H4 microplate reader.

### ImageJ quantitation of cells from slide-mounted sections

Image analyses and ImageJ quantification were performed from two areas of the retina around the optic nerve head (center retina) on retinal cross sections cut through the vertical meridian of the eye as shown by the boxed areas in the Fig. 3a. Three sections 70 μm apart were analyzed from each eye from each animal. The H&E or immunostained cells in each of the areas were counted individually by selecting the region of interest with the selection tool and using the Analyze Particles command of ImageJ. For ImageJ quantitation of immunofluorescence or immunoperoxidase staining, images were taken using the same exposure and digital gain setting to minimize differential background intensity. During quantitation, RGB images were converted to 32-bit grayscale images. For both cell counting and staining intensity measurement, the grayscale images were adjusted for the brightness and contrast to exclude noise pixels. Images were also adjusted for threshold to highlight all positive pixels to be measured. For cell counting, particle size and circularity were adjusted to exclude noise pixels, cellular fragments, processes, or tissue aggregates from counting. Same specifications were applied across the board for a given staining. Staining intensity was analyzed by measuring the integrated density using the measure tool in Image J. Background intensity was measured from four different areas of the images, averaged, and subtracted from the total intensity (integrated density) to obtain integrated density values for each image. The values from each measurement were averaged and expressed as mean ± SEM.

### Statistical analysis

Statistical analysis was performed with Graph Pad Prism 6.0 software (Graph Pad Software, La Jolla, CA, USA). Repeated measures analysis of variance (ANOVA) and Student’s *t* test were used for group comparisons. Bonferroni’s multiple-comparisons test was used to determine which ANOVA data sets were significantly different. A *p* value of less than 0.05 was regarded as statistically significant.

## Results

### Mild rTBI-induced neurodegeneration in the mouse brain

**Fig. 1:**
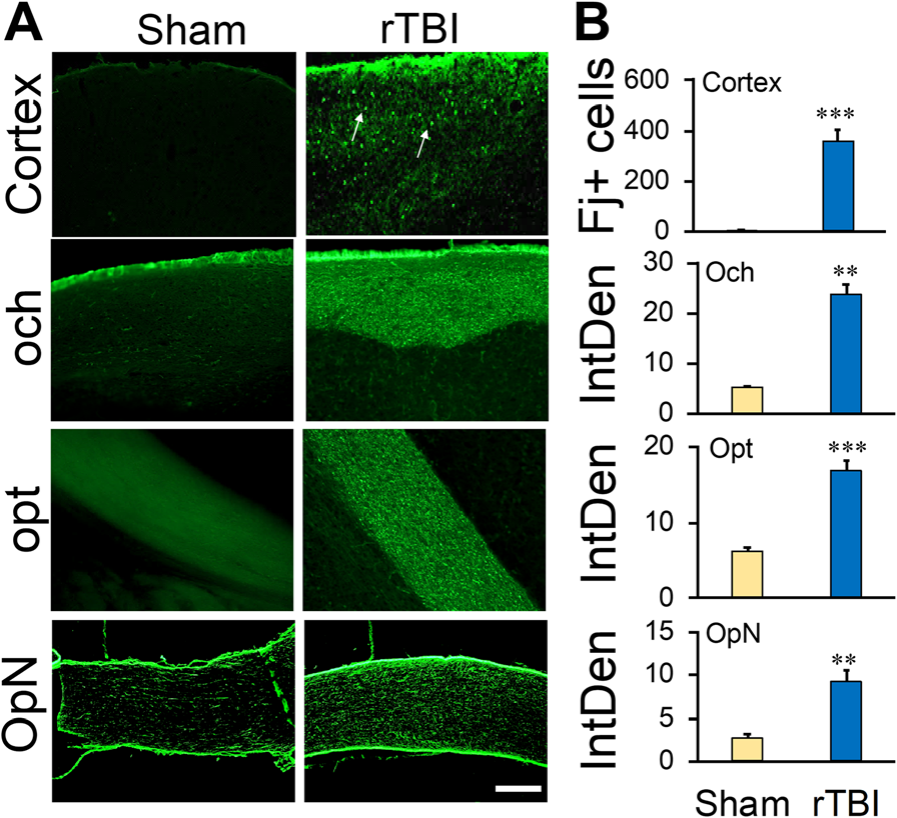
rTBI induces neurodegeneration in the brain of mice 7 days post-injury. **a** Representative fluorescence images showing fluoro-jade (FJ)-positive degenerating cells or fibers in different areas of the brain. Arrows indicate FJ-positive cells. Scale bar 50 μm. **b** Histograms showing the average number of FJ-positive cells or average FJ intensity in different brain areas. Values are shown as mean ± SEM. och, optic chiasm; opt, optic tract; OpN, optic nerve. **p < 0.001, ***p < 0.0001

A detailed investigation of the FJ-stained mouse brain sections at 7 dpi revealed FJ-positive cells in the cerebral cortex around the impact site in the rTBI mice as shown in Fig. 1. Consistent with the coordinates of impact delivery, major damage was observed in the parietal cortex on either side of the midline. FJ-positive cell bodies were observed in the cortex on either side of the midline (Fig. 1a, b). FJ-positive fibers and a few cell bodies were found in the optic chiasm, optic tracts, and optic nerves (Fig. 1a, b). Sham animals did not show any FJ-positive cells in any area of the brain or optic nerve. ImageJ quantitation revealed that there was a significant increase in neurodegeneration and nerve fiber damage in these areas (Fig. 1b).

### rTBI induced RGC loss and CCL20 expression in the retina of WT mice

**Fig. 2:**
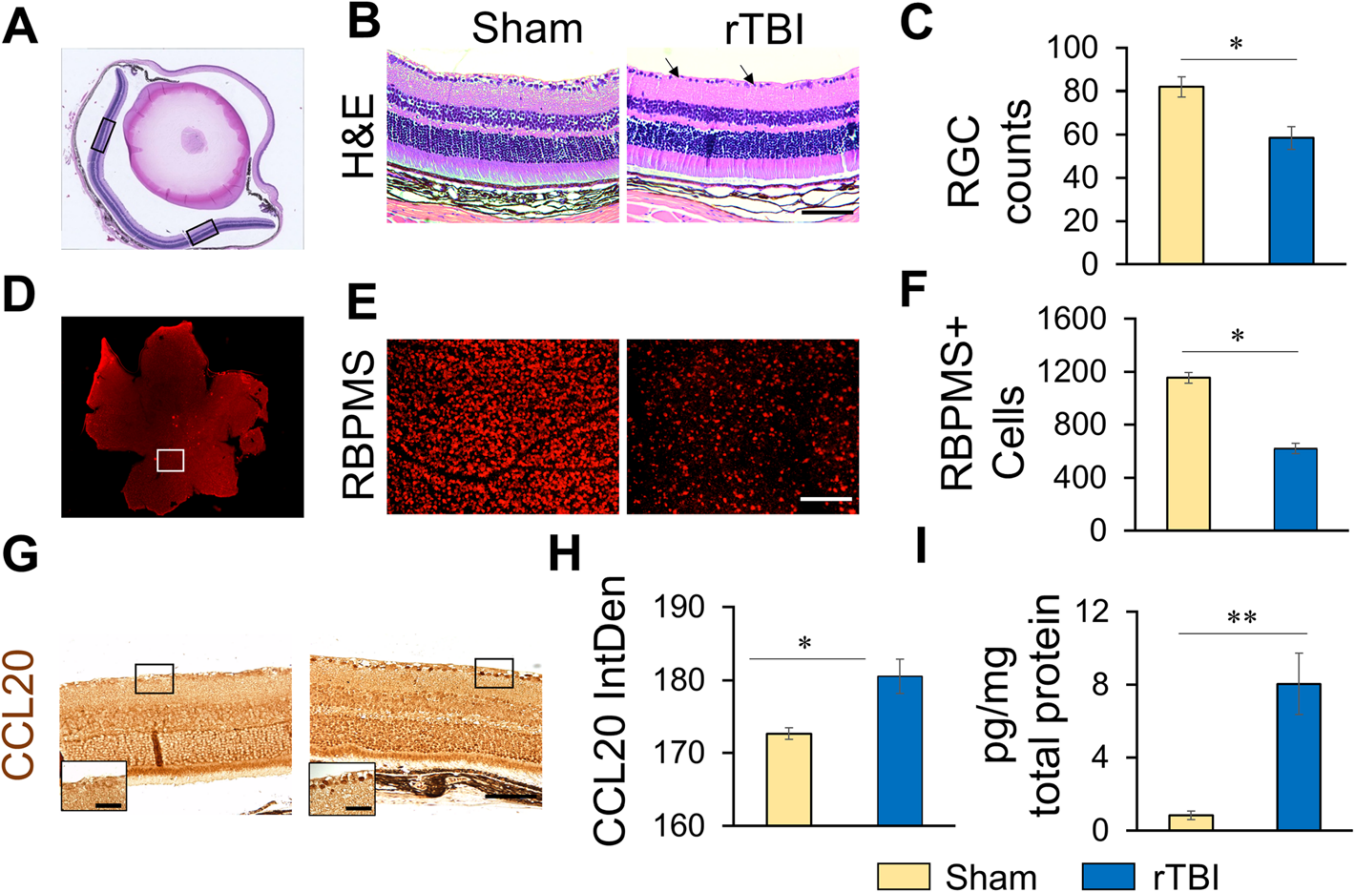
rTBI induces RGC loss and CCL20 expression in the retina 7 days post-injury. **a** Representative low magnification, bright field image of H&E staining of cross section of the whole eye. Images, as shown in **b** and **g**, are representations of one of the boxed areas in the **a**. Cells were counted or integrated density was measured  from both the boxed areas, and the average numbers were plotted as the histogram shown in **c** and **h** respectively. **b** Photomicrograph of H&E staining of retinal sections from one of the boxed areas in **a** under different experimental conditions. Arrows indicate RGC loss. **c** Histogram showing the RGC counts from the H&E-stained sections. **d** Retinal flat mount preparation showing immunostaining with anti-RBPMS antibody. **e** Representative high-magnification immunofluorescence images of the boxed area in **d** showing RBPMS-positive ganglionic cells under different experimental conditions. **f** Histogram showing RBPMS positive cell counts under different experimental conditions. **g** Photomicrograph showing immunoperoxidase staining with anti-CCL20 antibody of the retinal sections from one of the boxed areas shown in **a**. Scale bar 100 μm, inset, high-magnification image of the boxed area of the ganglionic cell layer, scale bar, 20 μm. **h** ImageJ quantitation of CCL20 immunoreactivity in the retina. **i** CCL20 protein expression in the retina as measured by Quantikine ELISA. *p < 0.01, **p < 0.001, ***p < 0.0001

H&E stained sections of retina showed decreased cellularity in the ganglion cell layer (GCL) of the retina of rTBI mice as compared to sham (Fig. 2a–c). Two areas of the retina on either side of the blind spot were selected to count the numbers of nuclei in the GCL. In the H&E-stained sections, cellular loss and presence of pyknotic cells were observed in the retina of the rTBI animals (Fig. 2b). ImageJ quantitation showed that in WT mice, the number of RGCs was reduced by ~29% following rTBI as compared to sham mice (Fig. 2c). To confirm this observation and eliminate any error due to regional variation of counting from the H&E sections, we specifically stained RGCs with anti-RBPMS antibody on retinal flat mount preparation (Fig. 2d, e). High-magnification images were taken using Keyence microscope from the retinal flat mount, and RGCs were counted from a collage using Keyence cell counting software as mentioned in the “Methods” section. RBPMS immunostaining of the whole retina also showed the RGC loss following rTBI (Fig. 2e, f). Immunoperoxidase staining using anti-CCL20 antibody showed significantly elevated inflammatory chemokine CCL20 expression in the retina, especially in the RGCs of rTBI mice compared to sham mice (Fig. 2g). ImageJ quantitation of CCL20 immunoreactivity showed significant increase in intensity in rTBI mice over sham mice (Fig. 2h). CCL20 ELISA from the retinal tissues also shows significant elevation of CCL20 protein expression (Fig. 2i). Together, these observations indicate an active inflammatory reaction in the retina following rTBI.

### rTBI failed to induce retinal pathologies and inflammation in CCR6−/− mice

**Fig. 3:**
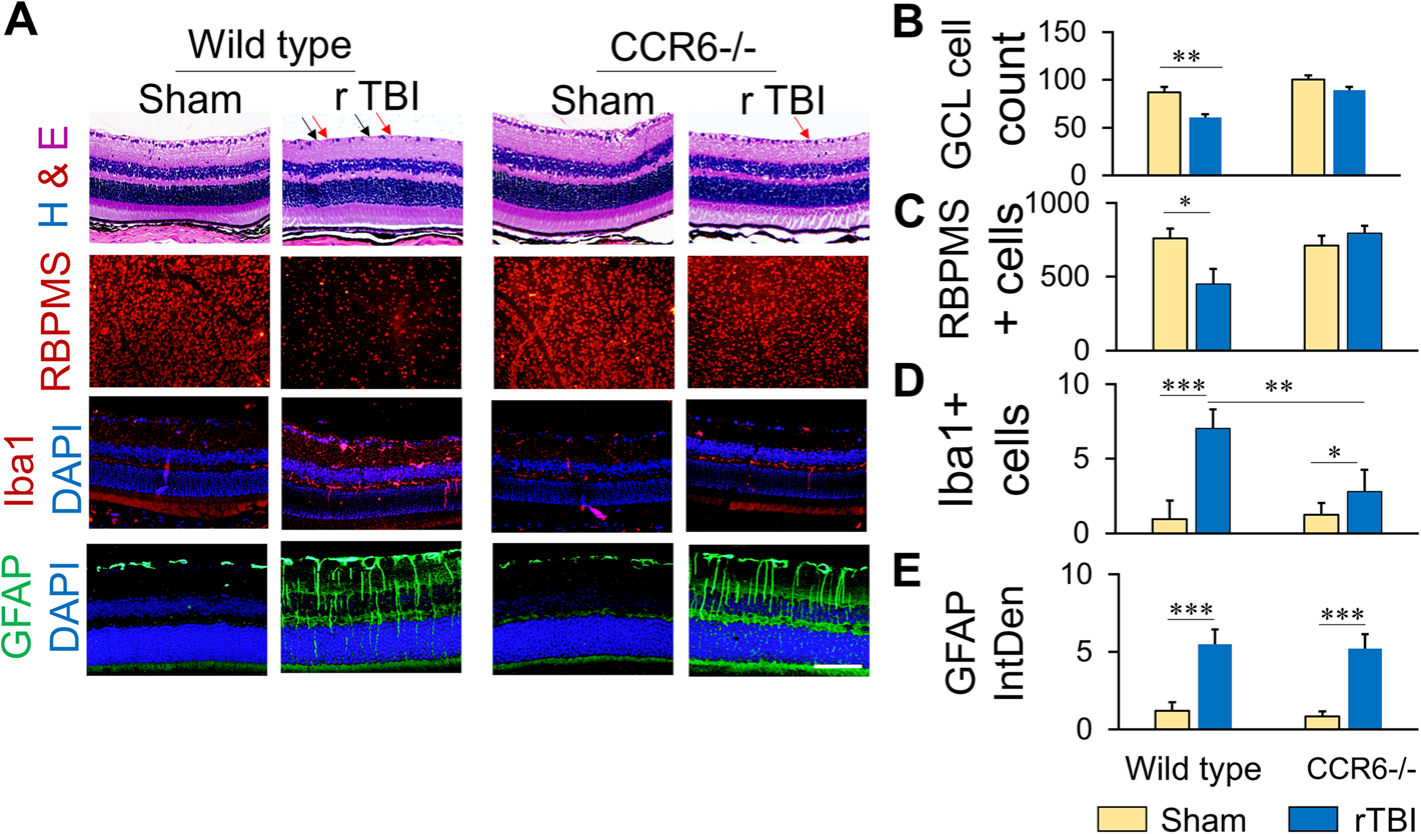
CCR6 knock down prevents retinal pathology and inflammation following rTBI. **a** High-magnification images of representative areas of the retina showing H&E staining, RBPMS-positive cells, Iba1-positive microglia, and GFAP-positive Müller cells in the retina of wild type or CCR6−/− mice. Ganglionic cells in the RGC layer is indicated by black arrows, and pyknotic cells are indicated by red arrows. Scale bar 100 μm. **b**–**e** Histograms showing cell count or intensity of immunoreactivity of ganglionic cell loss (**b**), RBPMS-positive ganglionic cells from the retinal whole mount preparation (**c**), Iba1-positive cells (**d**), and GFAP immunoreactivity (**e**). GCL, ganglion cell layer; RBPMS, RNA-binding protein with multiple splicing; GFAP, glial fibrillary acidic protein; Iba1, ionized calcium-binding adapter molecule 1. Values are shown as mean ± SEM. *p < 0.01, **p < 0.001, ***p < 0.0001

H&E staining of retinal sections and RBPMS staining of whole retina showed that rTBI induced cellular loss including RBPMS-positive RGC loss in the GCL in WT mice. However, in the CCR6−/− mice, rTBI failed to reduce the cellularity in the GCL as observed from the H&E sections (Fig. 3a, b). RBPMS-positive RGC counting from the flat mount preparation also indicated that rTBI significantly reduced RGC numbers in the retina as compared to sham in the WT mice but not in CCR6−/− mice (Fig. 3a, c).

Microglial activation is a key feature of the central nervous system (CNS) inflammation. Following rTBI, the number of Iba1-positive activated microglia significantly increased in the inner plexiform layer (IPL) of the retina in WT mice but not in CCR6−/− mice (Fig. 3a, d). ImageJ quantitation also showed the number of activated microglial (iba1-positive) cells in the WT rTBI group was significantly higher than the CCR6−/− group (Fig. 3d).

Müller cells, the major glial cell type of the retina, were activated following rTBI as evidenced by the upregulation of GFAP in the cell bodies and activated processes. ImageJ quantitation of GFAP immunoreactivity showed significant increase over sham animals. However, knocking out of CCR6 did not affect the Müller cell activation as indicated by GFAP upregulation after rTBI in CCR6−/− mice (Fig. 3a, e).

### PG treatment reduced CCL20 expression in the retina and improved rTBI induced retinal pathologies

**Fig. 4:**
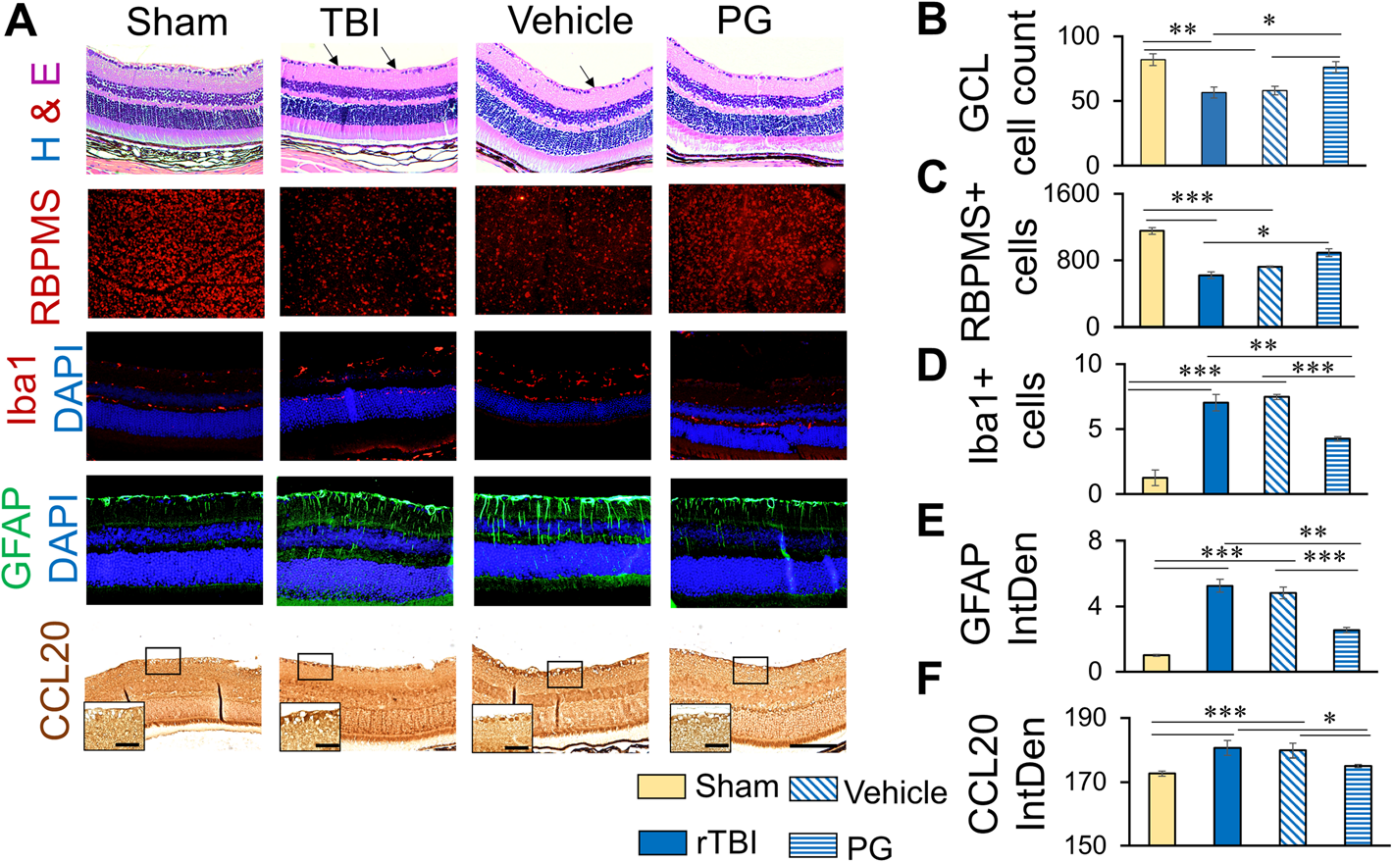
Pioglitazone treatment significantly reduces RGC loss, microgliosis, Müller cell activation, and CCL20 expression in the retina. **a** Representative immunofluorescent images showing RGC (H&E and RBPMS), Iba1 (red), GFAP (green), or CCL20 (bright field) immunoreactivity in the retina with or without PG treatment 7 days after rTBI. Scale bar 100 μm. Inset high magnification images of the boxed areas showing CCL20 expression in the retinal ganglionic cells, scale bar 20 μm. Blue fluorescence shows DAPI. Scale bar 100 μm. **b**–**f** Histograms showing average count of RGC (**b**, **c**), Iba1-positive cells (**d**), average fluorescence intensity of GFAP immunoreactivity (**e**), or CCL20 immunoreactivity (**f**) as quantitated by ImageJ. *p < 0.01, **p < 0.001, ***p < 0.0001.

PG is a widely used anti-diabetic drug with potent anti-inflammatory effect. In this study, we used PG as an anti-inflammatory drug with an aim to reduce CCL20 activity. We injected a single dose of 10 mg/kg PG, i.p. We optimized the dose in our pilot experiment and determined 10 mg/kg single dose as the optimum dose to be used in the rest of the study. As expected, CCL20 expression in the retina, especially in the GCL, was reduced after PG treatment to almost to the level of basal expression as observed in the sham animals (Fig. 4a, f). Moreover, PG treatment reduced retinal RGC loss. Both total cell count in the GCL from H&E staining and RBPMS-positive cell count from the retinal whole mount preparation showed significant recovery of cell loss following PG treatment indicating improvement (Fig. 4a–c). However, injections of the animals with equal volume of vehicle (40 μL per mouse) did not show any significant difference in RGC loss, GFAP, Iba1, or CCL20 immunoreactivity between rTBI and vehicle-treated groups (Fig. 4a, b).

Following rTBI, the number of Iba1-positive activated microglial cells significantly increased in the IPL of the retina in WT mice. PG treatment significantly reduced the number of Iba1-positive microglial cells in the retina 7 dpi (Fig. 4a, d). PG treatment significantly reduced the Müller cell activation as GFAP immunoreactivities significantly decreased in the retina following the treatment. The Müller cell processes which were distinctly visible become less visible following treatment showing resting Müller cell-like appearance (Fig. 4a, e).

### Treatment with anti-CCL20 neutralizing antibody reduced rTBI-induced pathologies in the retina

**Fig. 5:**
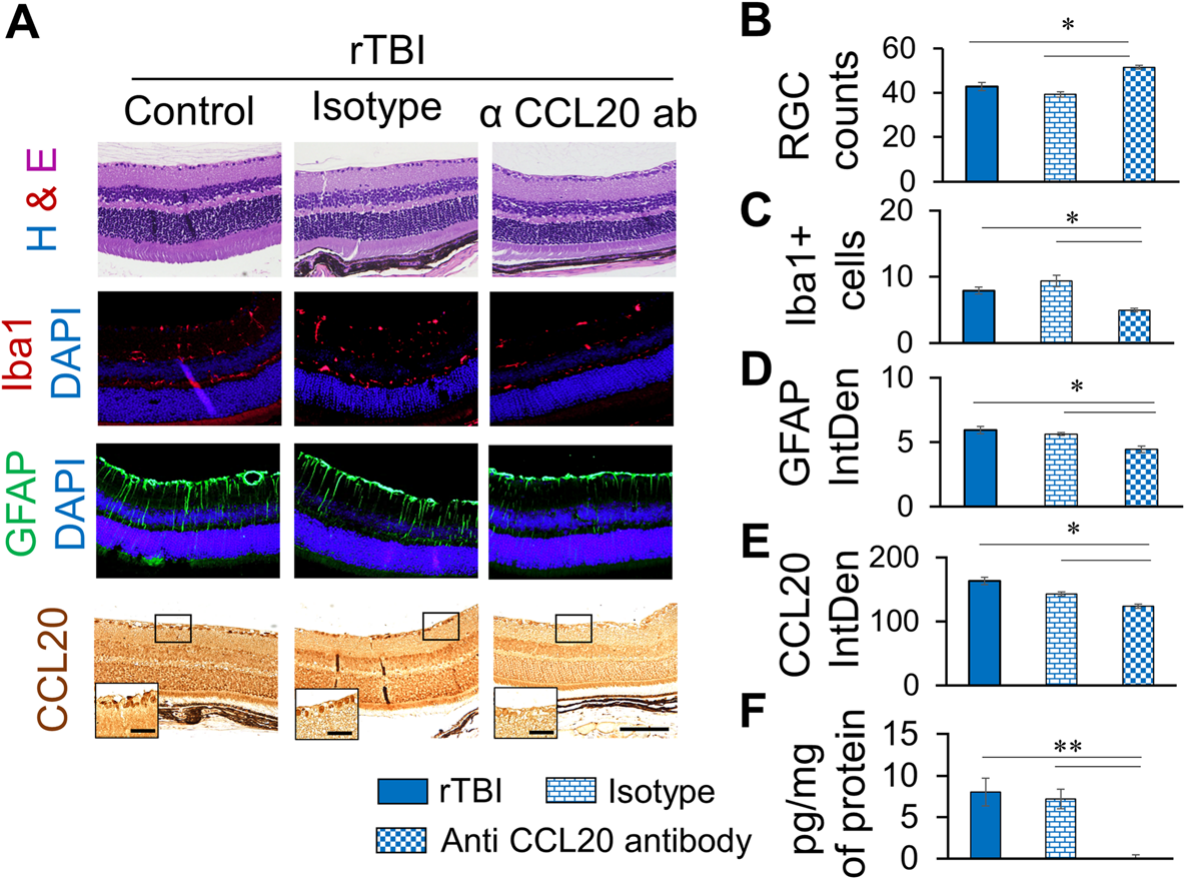
Anti-CCL20 antibody significantly reduces RGC loss, microgliosis, Müller cell activation and CCL20 expression in the retina. **a** Representative immunofluorescence images showing RGC (H&E), Iba1 (red), GFAP (green), or CCL20 (bright field) immunoreactivity in the retina with or without anti-CCL20 antibody or isotype treatment 7 days after rTBI. Scale bar 100 μm. Inset high-magnification images showing CCL20 expression in the retinal ganglionic cells, scale bar 20 μm. Blue fluorescence shows DAPI. **b**–**e** Histograms showing average count of RGC (**b**), Iba1-positive cells (**c**), average fluorescence intensity of GFAP immunoreactivity (**d**), or CCL20 immunoreactivity (**e**) as quantitated by ImageJ. **f** CCL20 protein expression in the retina as estimated by Quantikine ELISA. *p < 0.01, **p < 0.001, ***p < 0.0001

To determine whether CCL20 was indeed involved in the induction of TBI pathologies in this model or not, we treated a subset of mice with anti-CCL20 antibody or isotype IgG. Control rTBI and isotype-treated rTBI mice showed loss of RGC, microglial activation, Müller cell activation, and CCL20 expression in the retina. On the other hand, these pathological and inflammatory changes were significantly reduced in the mice treated with anti-CCL20 antibody. When compared with rTBI or isotype IgG-treated rTBI mice, the number of RCG count increased in the anti-CCL20 antibody-treated rTBI mice (Fig. 5a, b). Also, Iba1-positive cells decreased (Fig. 5a, c), Müller cell activation decreased (Fig. 5a, d), and CCL20 immunoreactivity in the retina, especially in the RGCs, decreased in the anti-CCL20 antibody-treated rTBI mice (Fig. 5a, e). This observation was supported by ELISA which showed that CCL20 protein expression in retina significantly decreased in mice treated with anti-CCL20 antibody (Fig. 5f). Together, these observations clearly indicate that in vivo neutralization of CCL20 improved the rTBI-induced pathologies and inflammation in the retina.

### rTBI did not induce microglial activation in the optic nerve in CCR6−/− mice or following CCL20 antibody treatment in the wild-type mice

**Fig. 6:**
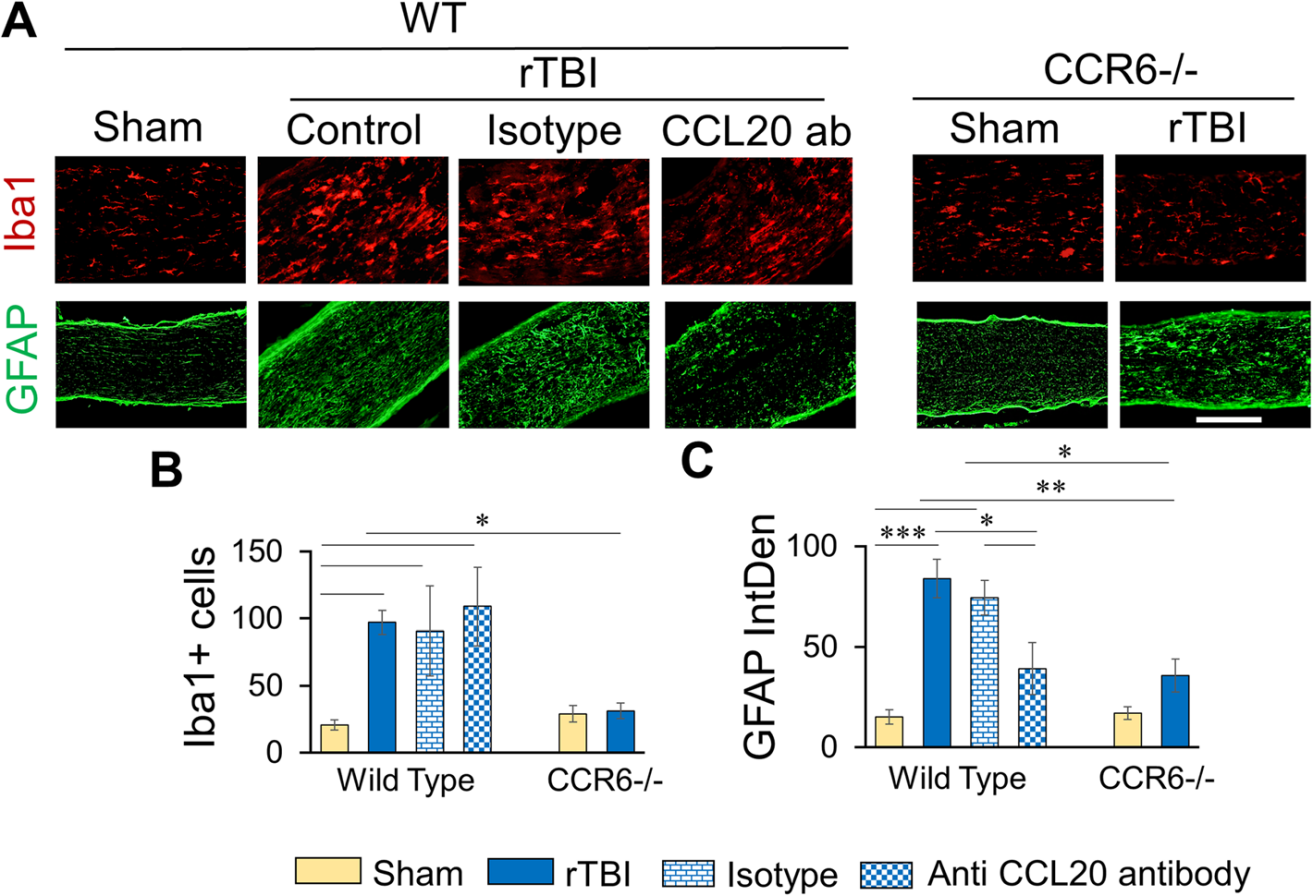
Effect of anti-CCL20 antibody or CCR6 knock down on microgliosis and astrogliosis in optic nerve following rTBI. **a** Representative immunofluorescence images showing Iba1 (red) or GFAP (green) immunoreactivity in the optic nerve. Scale bar 100 μm. **b**, **c** Histograms showing average count of Iba1-positive cells (**b**) or average fluorescence intensity of GFAP immunoreactivity (**c**) as quantitated by ImageJ. Values are shown as mean ± SEM. *p < 0.01, **p < 0.001, ***p < 0.0001

Microglial activation was observed in the optic nerve after rTBI in the WT mice but not in the CCR6−/− mice. The number of Iba1-positive cells significantly increased in WT rTBI group as compared to WT sham (Fig. 6a, b). The activated microglial cells were also enlarged in the rTBI group. However, this change was absent in the CCR6−/− group.

On the other hand, astrocytes were activated after rTBI in the optic nerve in both WT and CCR6−/− groups as compared to the respective sham animals. There was a significant difference in GFAP expression between sham and rTBI groups of both WT and CCR6−/− animals, but no difference in GFAP expression was observed between WT rTBI and CCR6−/− rTBI animals (Fig. 6a, c).

Following anti-CCl20 antibody treatment, rTBI did not change the microglial response (Fig. 6a, b) while the astrocyte activation was decreased in these mice (Fig. 6a, c). The Iba1-positive cell counts remained the same in all three groups (Fig. 6a, b) while GFAP immunoreactivity significantly reduced after rTBI (Fig. 6a, c).

## Discussion

In this study, we used an established mouse model of mild rTBI without skull fracture and penetrating wounds. We observed FJ-positive cells in the impact area 7 days after the final impact. FJ positivity of the optic tract and optic chiasm indicated that these areas were also affected by the impact. We observed significant damage and inflammation in the retina. The decrease in retinal cellularity, presence of pyknotic cells, and decrease in RBPMS-positive ganglion cells in the retina of rTBI mice clearly indicate that rTBI caused subtle degenerative changes in the retina. Comparable observations have been reported by Tzekov et al. in a very similar model of rTBI in mice [7], although they observed less RGC loss which probably resulted from the lower impact depth (1 mm) used in their model compared to the present one (1.5 mm). In addition, we have observed nerve fiber damage in the optic tract, optic chiasm, and optic nerve in this model. Previously, Tzekov et al. correlated retinal damage following rTBI directly to the optic nerve fiber damage and reduction following rTBI in mice [8]. To our knowledge, this is the first report showing degenerative changes in the entire visual pathway from the retina to the cortex in closed head rTBI model as early as 7 dpi.

Although the role of microglia in neurodegeneration is controversial, it is clear that these cells play an important role in maintaining neuronal homeostasis [46, 47]. They are inflammatory response mediators [48], and their hyperactivation has been implicated in damaging the target tissue [49]. Suppression of microglia improves axonal regeneration and RGC survival after axotomy [50]. Recently, it has been shown that activated microglia cleared the dead RGC somas from the retina following axotomy [51]. In this study, we have also observed profound microglial activation in both the retina and optic nerve. The Iba1 immunoreactivity of activated microglia with amoeboid morphology increased in the retina and optic nerve in WT mice 7 days after rTBI indicating the inflammatory status of these regions.

Müller cells are the type of retinal glial cells involved in neuronal homeostasis, maintaining structural and functional integrity and degrading foreign materials. These cells provide necessary support to retinal neuronal cell types. Under physiological conditions, the cell bodies of Müller cells show a low basal level of GFAP production. Studies have shown strong upregulation of GFAP immunoreactivity in the retina in a blast injury model 7 dpi [52, 53]. Mammadova et al. reported upregulation of GFAP immunoreactivity 30 dpi [54]. Glial cell activation immediately after retinal injury is important for protection of the retinal neurons. Müller cells may exacerbate retinal degeneration in diabetic neuropathy and other retinal degenerative diseases [55, 56]. In our study, activation of Müller cells clearly indicates disturbance in the neuronal homeostasis and inflammatory reactions in the retina in response to rTBI. The exact role played by these cells following injury in our model needs further investigation.

CCL20 is a pro-inflammatory chemokine which interacts specifically with its sole receptor CCR6 expressed on Th17 and T_reg_ cells [37] and induces chemotaxis of DCs, T cells, and B cells [20]. CCL20-CCR6 interaction modulates inflammatory reaction in the target tissue. In the eye, the cornea is known to produce high basal level of CCL20 which attracts CCR6-expressing Th17 cells under physiological condition and the expression increases under inflammatory conditions [57, 58]. Involvement of CCR6 in the development of DED by recruiting Th17 cells has been shown by Dohlman et al. [28]. Patients suffering from AMD have shown higher levels of Th1 and Th17 cells promoting M1 macrophage differentiation [59], and Singh et al. have shown that Th17 cells were involved in AMD-induced retinal damage and these cells were also CCR6-positive [38]. Importance of CCL20-dependent influx of IL17+ CCR6+ IL22+ γδT cells in restoring the epithelial cells and nerve fibers in the eye was shown by Li et al. [60]. Interaction between CCL20-CCR6 is interesting because they display both homeostasis and activation of the immune system, and this dual characteristic is suggestive of CCR6 being involved in the priming phase of the immune response [61]. Liston et al. have reported that inhibition of CCR6 reduced the severity of experimental autoimmune encephalomyelitis (EAE) by priming autoreactive lymphocytes [61]. Thus, the importance of CCR6 in mediating CCl20-induced degeneration of the target tissues has been shown. In this study, we also observed the involvement of CCL20 in retinal damage following rTBI. To our knowledge, we are the first to report the increased expression of CCL20 in the retina following closed head rTBI in mice and this novel finding led us to investigate the involvement of CCR6 in this process. With this objective, we induced rTBI in CCR6−/− mice. In these mice, RGC loss and microglial activation were reduced significantly. Infiltration of neutrophil and T cells in the retina was also observed following rTBI, and this response was blocked by anti-CCL20 antibody treatment (Additional file 1: Figure S1). This needs further investigation to establish the immune cell response in the retina following rTBI. In the optic nerve also, knock down of CCR6 blunted the microglial response to rTBI. However, astrocyte activation was not altered in the CCR6−/−mice as compared to sham, indicating a CCR6-independent mechanism in this phenomenon that needs further investigation.

We have previously shown that CCL20 is expressed in the spleen and brain and plays a major role in the inflammatory reaction in the brain after TBI [29]. Hu et al. reported the involvement of this chemokine in neurodegeneration in spinal cord injury [32]. A recent study identified CCL20 as a dual-acting chemokine with the potential for inhibiting immune reactions and more importantly for attracting inflammatory effectors and activators [58]. CCL20 gene expression in the mouse retina following experimental diabetic retinopathy has been reported by Rossi et al. [39]. In the present study, we have observed CCL20 expression in the retina following rTBI. To elucidate the role played by CCL20 in rTBI-induced retinal pathologies, we pharmacologically blocked CCL20 using PG. PG and other PPARγ agonists have been shown to downregulate CCL20 [41, 57]. PG is used as an anti-diabetic drug and has recently been shown to be cardio-protective [62]. By using single i.p. dose of PG, we were able to significantly reduce CCL20 expression in the retina, especially in GCL following rTBI. PG treatment also reduced cellular loss in GCL, indicating its neuroprotective role. Microglial activation and GFAP expression in the retina were reduced in PG-treated animals indicating overall reduction of inflammation. PPARγ activation by itself is neuroprotective and neurorestorative [63]. It may exploit different pathways in reducing neuroinflammation [63, 64] including NF-κB pathway [65], of which CCL20 is an important mediator. The observations in this study indicate that CCL20 is an important player in the entire mechanism. Since PG is a wide-spectrum anti-inflammatory drug affecting multiple inflammatory mediators, to fully elucidate the involvement of CCL20, in this study we treated the mice with anti-CCL20 antibody following rTBI. The results of CCL20 neutralization indicate that the degenerative and inflammatory effects seen in this study were mediated, at least in part, by CCL20.

## Conclusion

rTBI induces damage of the visual pathway in mice. RGC loss in the retina, microglial activation, and Müller cell activation in the retina clearly show active inflammation in the eye 7 days post rTBI. The significant reduction of RGC loss and neuroinflammation in CCR6−/− mice or PG treatment indicate that CCL20 could have a role in this mechanism. This was further verified by the reduction of RGC loss, microglial activation, and Müller cell activation after anti-CCL20 antibody treatment following rTBI. CCR6 knockout renders significant protection against rTBI-induced retinal damage and neuroinflammation. Further studies are necessary to elucidate the specific role of the CCL20-CCR6 axis in TVD.

## Additional file

Additional file 1: **Figure S1.** Cellular infiltration in the retina. Immunostaining with anti-CD3 (1:100) or anti-MPO (myeloperoxidase) antibody (1:250) indicates infiltration of a few CD3-positive T cells or MPO-positive neutrophils in the rTBI or isotype-treated rTBI mouse retina. These cells were not observed in sham or anti-CCL20 antibody-treated animals. Upper panel showing immunofluorescent images of CD3-positive cells (white arrows). Inset high-magnification image of the cell and the lower panel shows the bright field images of MPO (black arrows). Scale bar 100 μm. MPO, myeloperoxidase. (TIF 4186 kb)

## Abbreviations

AMD: Age-related macular degeneration
CCL20: C-C chemokine ligand 20
CCR6: C-C chemokine receptor 6
CCR6−/−: CCR6 knock out
DAB: Diaminobenzidine
DAPI: 4′,6-Diamidine-2′-phenylindole dihydrochloride
DED: Dry eye disease
DPX: Distyrene-tricresyl phosphate-xylene
GCL: Ganglion cell layer
GCS: Glasgow Coma Scale
GFAP: Glial fibrillary acidic protein
H&E: Hematoxylin and eosin
Iba1: Ionized calcium binding adaptor molecule
PBS: Phosphate-buffered saline
PG: Pioglitazone
PPARγ: Peroxysome proliferator-activated receptor gamma
RBPMS: RNA binding protein with multiple splicing
RGC: Retinal ganglion cells
rTBI: Repeated traumatic brain injury
TBI: Traumatic brain injury
TVD: Repeated traumatic brain injury-induced visual dysfunction
WT: Wild type
