# Supplementary figures and images for: CCL20-CCR6 axis modulated traumatic brain injury-induced visual pathologies
Source: J Neuroinflammation. 2019 May 31;16:115. doi: 10.1186/s12974-019-1499-z (PMC6544928; doi:10.1186/s12974-019-1499-z)

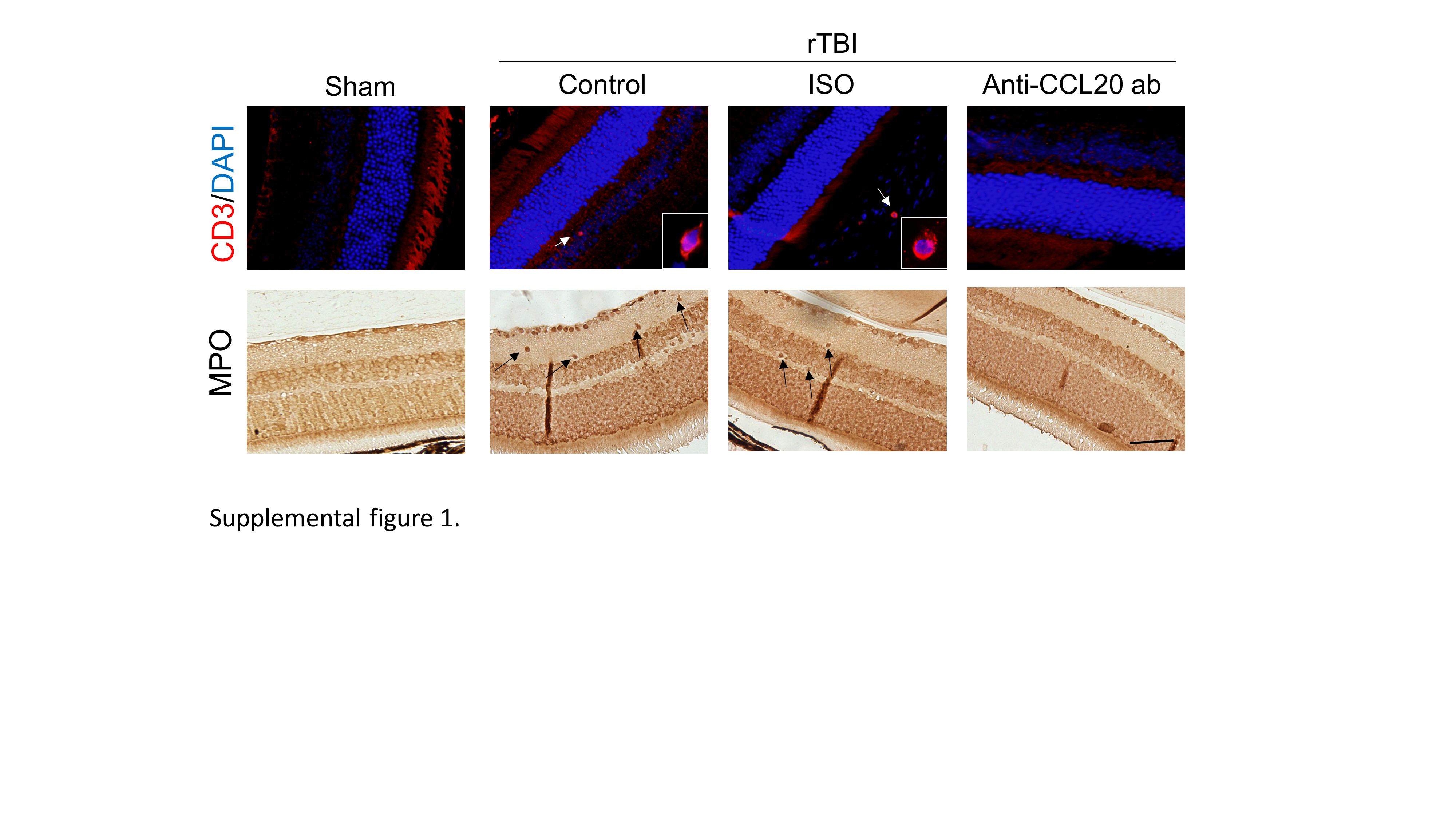

Supplement: Supplementary file 1 — Figure S1. Cellular infiltration in the retina. Immunostaining with anti-CD3 (1:100) or anti-MPO (myeloperoxidase) antibody (1:250) indicates infiltration of a few CD3-positive T cells or MPO-positive neutrophils in the rTBI or isotype-treated rTBI mouse retina. These cells were not observed in sham or anti-CCL20 antibody-treated animals. Upper panel showing immunofluorescent images of CD3-positive cells (white arrows). Inset high-magnification image of the cell and the lower panel shows the bright field images of MPO (black arrows). Scale bar 100 μm. MPO, myeloperoxidase. (TIF 4186 kb) [file 12974_2019_1499_MOESM1_ESM.tif]
